# Strong coupling between WS_2_ monolayer excitons and a hybrid plasmon polariton at room temperature

**DOI:** 10.1515/nanoph-2024-0021

**Published:** 2024-04-15

**Authors:** Yuhao Zhang, Hans-Joachim Schill, Stephan Irsen, Stefan Linden

**Affiliations:** Physikalisches Institut, Rheinische Friedrich-Wilhelms-Universität Bonn, 53115 Bonn, Germany; Electron Microscopy and Analytics, Center of Advanced European Studies and Research (Caesar), 53175 Bonn, Germany

**Keywords:** strong light–matter coupling, TMDC monolayer, excitons, surface plasmon polaritons, silver nanogratings

## Abstract

Light–matter interactions between plasmonic and excitonic modes have attracted considerable interest in recent years. A major challenge in achieving strong coupling is the identification of suitable metallic nanostructures that combine tight field confinement with sufficiently low losses. Here, we report on a room-temperature study on the interaction of tungsten disulfide (WS_2_) monolayer excitons with a hybrid plasmon polariton (HPP) mode supported by nanogroove grating structures milled into single-crystalline silver flakes. By engineering the depth of the nanogroove grating, we can change the character of the HPP mode from propagating surface plasmon polariton-like (SPP-like) to localized surface plasmon resonance-like (LSPR-like). Using reflection spectroscopy, we demonstrate strong coupling with a Rabi splitting of 68 meV between the WS_2_ monolayer excitons and the lower HPP branch for an optimized nanograting configuration with 60 nm deep nanogrooves. In contrast, only weak coupling between the constituents is observed for shallower and deeper nanogratings since either the field confinement provided by the HPP is not sufficient or the damping is too large. The possibility to balance the field confinement and losses render nanogroove grating structures an attractive platform for future applications.

## 1 Introduction

Composite structures formed by coupling a photonic micro/nanoresonator with an optical material supporting a strong exciton resonance provide unique capabilities to investigate and engineer light–matter interactions in solid-state systems [1], [2], [3], [4], [5], [6]. The character of the interaction is largely determined by the ratio of the energy exchange rate between light and matter, i.e., the Rabi frequency, and the average dissipation rate. In the weak coupling regime, the Rabi frequency is smaller than the average dissipation rate and the eigenstates of the structure can still be described in terms of the exciton and photon modes. Modifications of the local photonic density of states by the photonic structure can, however, alter the radiative life-time of the emitters [7], [8], [9], [10]. A qualitatively different situation arises in the strong coupling regime, where the Rabi frequency exceeds the dissipation rate and the energy is coherently exchanged between the light field and the emitters. This interaction leads to the formation of new polaritonic eigenmodes with light and matter character [1]. In the spectral domain, strong coupling manifests itself by an avoided crossing of the branches of the polaritonic eigenmodes [3], [4], [5].

Achieving strong light–matter interactions requires a meticulous selection of both the material system and the photonic structure. With regard to the material properties, the key requirements are an exciton state with large oscillator strength and low nonradiative damping rate. Moreover, the material should be robust and easy to combine with a wide range of photonic structures. Monolayers of transition metal dichalcogenides (TMDCs) such as tungsten disulfide (WS_2_) meet these requirements [11]. TMDC monolayers are atomically thin semiconductors with a direct band gap. Their two-dimensional character in conjunction with the reduced screening by the dielectric environment results in exciton states with large oscillator strength and a binding energy of several hundred meV [12], [13], [14]. The latter aspect renders TMDC monolayers an attractive material class for light–matter investigations at room temperature. Strong coupling of TMDC monolayer excitons and dielectric resonator [15], [16], [17], [18], [19], [20], [21] and grating structures [22] as well as different plasmonic nanocavities [23], [24], [25], [26], [27] has been achieved.

In terms of the photonic properties, the design of the structure must offer a suitable trade-off between the achievable vacuum field strength and losses. For instance, metallic nanostructures supporting localized surface plasmon resonances (LSPR) can confine light in nanometric volumes [28], [29], [30], [31], [32]. However, this typically comes at the cost of significant absorptive and radiative losses. In comparison, surface plasmon polaritons (SPP) propagating at the interface between a metal and a dielectric material feature lower losses but also a weaker confinement of the electromagnetic near-field limiting the vacuum field strength [33], [34]. Simultaneous optimization of the vacuum field strength and the damping is thus challenging for pure LSPR and SPP modes. Ideally, one would like to utilize a plasmonic structure that combines the best features of these two types of modes. Metallic nanogratings can host hybrid plasmon polaritons (HPPs) that result from the strong coupling of LSPRs and SPPs [35], [36], [37], [38], [39], [40]. By varying the grating geometry, the character of the HPP branches can be tuned for a given frequency from LSPR-like to SPP-like [38], [39]. This offers a promising approach to engineer metallic nanostructures for strong light–matter interactions that combine strong local field enhancement with sufficiently low damping.

**Figure 1:**
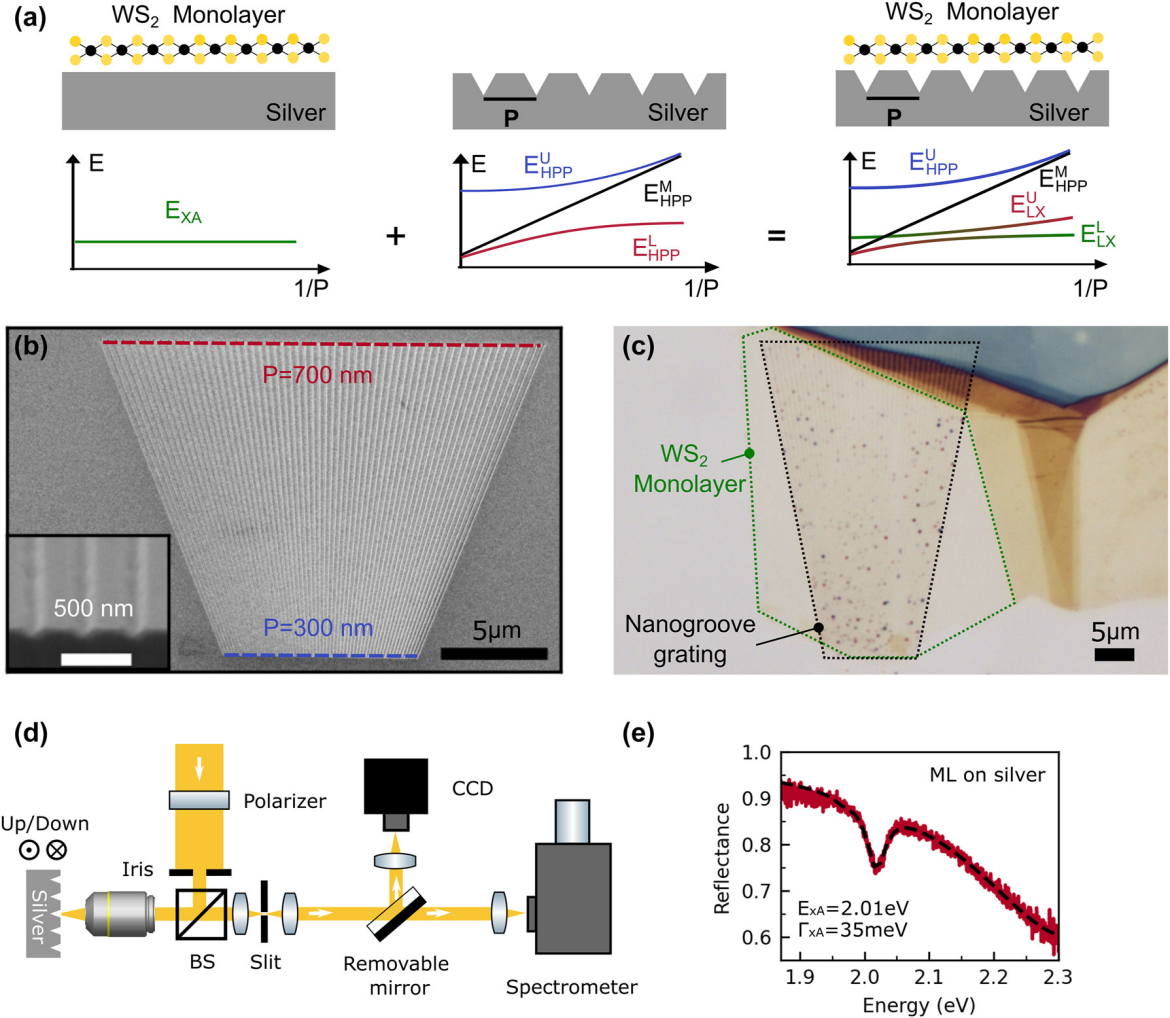
Sample design and experimental setup. (a) Scheme of the three structures studied in this article. Left panel: WS_2_ monolayer deposited on top of a single-crystalline silver flake. Middle panel: Nanogroove grating with period *P* milled into a single-crystalline silver flake. Right panel: Composite structure consisting of a WS_2_ monolayer deposited on top of a nanogroove grating with period *P* milled into a single-crystalline silver flake. For optimized geometry parameters, the lower branch of the HPP of the nanogroove grating strongly couples with the A-exciton resonance of the monolayer resulting in an avoided crossing of the two modes. (b) Scanning electron micrograph of a fan-shaped nanogroove grating structure taken at a tilt angle of 36°. The inset shows a cross section of the grating structure. (c) Optical micrograph of a WS_2_ monolayer deposited on a nanogroove grating structure. The outlines of the monolayer and the grating are marked by the green and black dashed curve, respectively. (d) Scheme of the white light reflectance spectroscopy setup. (e) Reflection spectrum of a WS_2_ monolayer deposited on a silver flake.

Here, we report on a room-temperature study on the interaction of TMDC monolayer excitons with a HPP mode. For this purpose, we deposit WS_2_ monolayers on nanogroove grating structures milled into single-crystalline silver flakes (see Figure 1). By increasing the depth of the nanogrooves, we vary the LSPR and SPP fractions of the lower HPP branch at the energy of the WS_2_ monolayer A-exciton mode. Using reflection spectroscopy, we demonstrate that strong coupling between the WS_2_ monolayer exciton mode and the lower HPP branch can be achieved for optimized nanogroove geometry parameters. Our results demonstrate the potential to tailor light–matter interactions in composite structures with a HPP mode.

## 2 Results and discussion

The composite structures were prepared according to the following procedure (see Methods for details). In the first step, single-crystalline silver flakes with a typical lateral size of hundreds of microns and a thickness of several microns were grown on a silicon substrate using an ammonium hydroxide-controlled polyol reduction process [41]. Next, focused ion beam milling was used to define fan-shaped nanogroove gratings in selected silver flakes (see Figure 1(b)). Within a grating structure, the period *P* varies continuously from 300 nm (bottom) to 700 nm (top). The nanogrooves have a Gaussian-like shape with a width of approximately 100 nm. The depth *D* of the nanogrooves was controlled by the milling time. Finally, atomically thin WS_2_ monolayers were prepared by a mechanical exfoliation method [42] and transferred onto the different nanogroove gratings. An optical micrograph of a completed sample is shown in Figure 1(c).

The samples were optically characterized with a home-built white light reflectance spectroscopy setup (see Figure 1(d)). A halogen light bulb served as the light source. After reflection from a 50:50 beam splitter, the light was focused with a microscope lens (Mitutoyo M Plan Apo 50 NA = 0.55) onto the sample. An iris diaphragm placed in front of the beam splitter was used to reduce the numerical aperture of the illumination to lower than 0.2. The light reflected from the sample was collected with the same microscope lens and passed through the beam splitter. An adjustable slit placed in an intermediate image plane served as a spatial filter to select the portion of the reflected light from the fan-shaped grating corresponding to a specific grating period. Reflection spectra for different grating periods were measured by shifting the fan-shaped grating structure along the nanogroove direction up or down. More specifically, we started at the bottom part of the grating where *P* = 300 nm. Subsequently, the grating was vertically moved in the steps of 1 μm resulting in a variation of the grating period by Δ*P* = 10 nm. The spectrum of the light was recorded with a cooled CCD camera (Princton Instruments PIXIS 256) attached to a spectrometer (Princton instruments ACTON SP 2300).

Before we address the composite structures, it is instructive to consider the optical properties of a WS_2_ monolayer and the silver nanogroove gratings separately. We start with the WS_2_ monolayer. Figure 1(e) depicts the room temperature reflection spectrum of a WS_2_ monolayer deposited on a planar silver flake. The spectrum is normalized with respect to the reflectance of the bare silver flake. The spectrum features a pronounced dip at *E*_xA_ = 2.01 eV. This resonance can be attributed to the so-called A-exciton of the WS_2_ monolayer, which is connected to direct gap transitions with lower energy at the K point [12]. The full width at half maximum (FWHM) linewidth of the exciton resonance as obtained by a Lorentzian-lineshape fit is Γ_xA_ = 35 meV, in line with recent reports on the exciton linewidth of a WS_2_ monolayer coupled to a silver film [26]. In comparison, the room temperature linewidth of WS_2_ monolayers deposited on planar dielectric substrates is in the order of 25 meV [43], indicating that the direct contact of the WS_2_ monolayer with the single crystalline silver crystal only results in a moderate line broadening.

Next, we consider silver nanogroove gratings and the plasmonic modes supported by such structures. Each nanogroove constitutes a nanoscale cavity that can host a LSPR. Its resonance energy *E*_LSPR_ depends on the nanogroove geometry. For instance, increasing the nanogroove depth leads to a redshift of the resonance [26]. If the nanogrooves are arranged in a periodic array, they form a grating coupler structure that allows to resonantly excite SPPs with an incident TM-polarized plane wave. For normal incidence, the wave vectors of the excited SPPs follow from the phase matching condition $k_{\text{SPP}}=\pm n\frac{2\pi }{P}\hat{x}$ , where *P* is the grating period, $\hat{x}$ is the in-plane unit vector perpendicular to the nanogrooves, and *n* is a positive integer.

LSPRs and SPPs have different spectral characteristics. SPPs at a flat silver–air interface exhibit an almost linear dispersion relation *E*_SPP_(***k***_SPP_) in the visible spectral range [44]. In contrast, the LSPR of the nanogrooves is a dispersionless mode. Furthermore, the FWHM linewidth Γ_LSPR_ of the nanogroove LSPR is expected to be significantly larger than the FWHM linewidth Γ_SPP_ of the SPP mode [38].

For suitable geometry parameters, the LSPR mode strongly interacts with the forward (^+^) and backward (^−^) propagating SPPs resulting in the formation of HPP modes [40]. The energy eigenvalues *E*_HPP_ of the latter can be calculated using a coupled oscillator model $\hat{H}{\left[\alpha^{+},\beta ,\alpha^{-}\right]}^{T}=E_{\text{HPP}}{\left[\alpha^{+},\beta ,\alpha^{-}\right]}^{T}$ , where the Hamiltonian of the coupled system is given by

$$\hat{H}=\left(\begin{array}{ccc} E_{\text{SPP}}^{+}-ı\frac{\Gamma_{\text{SPP}}^{+}}{2} & g & 0\\ g & E_{\text{LSPR}}-ı\frac{\Gamma_{\text{LSPR}}}{2} & g\\ 0 & g & E_{\text{SPP}}^{-}-ı\frac{\Gamma_{\text{SPP}}^{-}}{2} \end{array}\right).$$

Here, *g* is the coupling strength between the LSPR mode and the SPP modes. A direct coupling between the forward and backward moving SPPs is neglected in the model. The Hopfield coefficients *α* ^+^, *α* ^−^, and *β* specify the SPP^+^, SPP^−^, and LSPR mode fractions, respectively, in the HPP modes, with |*α* ^+^|^2^ + |*α* ^−^|^2^ + |*β*|^2^ = 1. For normal incidence, the excited SPPs are energetically degenerate, i.e., the condition $E_{\text{SPP}}^{+}=E_{\text{SPP}}^{-}\equiv E_{\text{SPP}}$ holds. Since the coupling strength *g* is considerably larger than the FWHM linewidths Γ_LSPR_ and Γ_SPP_, we can neglect the influence of the damping when calculating the real energy eigenvalues of the upper, lower, and middle branch of the HPP:

$$\begin{array}{ll} E_{\text{HPP}}^{\text{U,L}} & =\frac{E_{\text{SPP}}+E_{\text{LSPR}}}{2}\pm \frac{1}{2}\Delta E_{\text{UL}},\\ E_{\text{HPP}}^{\text{M}} & =E_{\text{SPP}}. \end{array}$$

Here, $\Delta E_{\text{UL}}\left(\delta \right)=\sqrt{\delta^{2}+8g^{2}}$ is the energy splitting between the upper and lower branch, while *δ* = *E*_SPP_ − *E*_LSPR_ is the detuning between the SPP and LSPR modes. For zero detuning, the energy splitting is given by $\Delta E_{\text{UL}}\left(0\right)=2\sqrt{2}g$ . The FWHM linewidths of the polariton branches can be calculated with the corresponding Hopfield coefficients to Γ_HPP_ = |*α* ^+^|^2^Γ_SPP_ + |*α* ^−^|^2^Γ_SPP_ + |*β*|^2^Γ_LSPR_.

**Figure 2:**
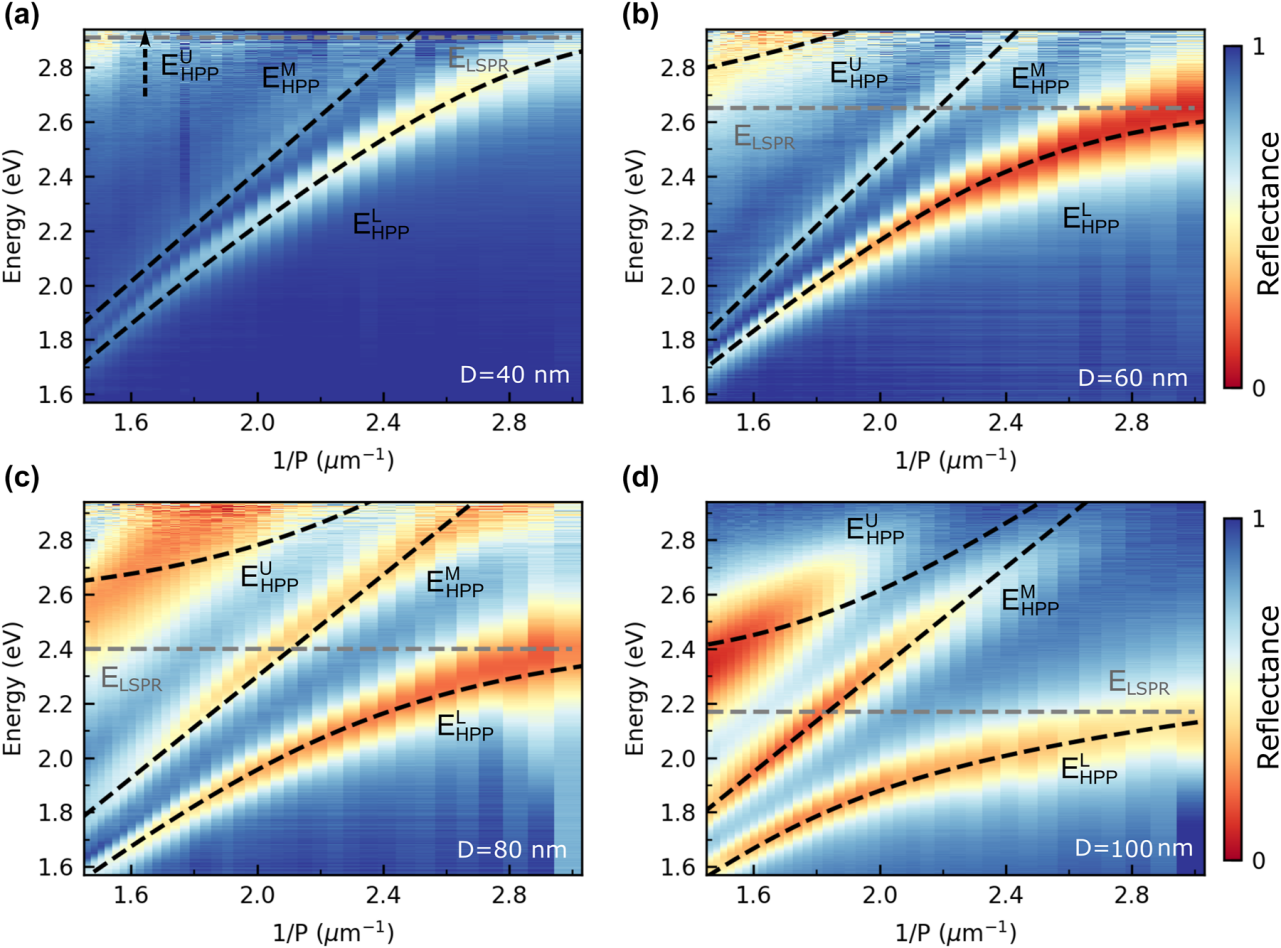
Reflectance spectra of bare silver nanogroove grating structures. (a)–(d) Color-coded normal incidence reflection spectra of nanogroove grating structures with depths *D* of 40 nm, 60 nm, 80 nm, and 100 nm, respectively, recorded for TM polarized light and plotted versus the inverse grating period 1/*P*. The dashed black curves in each case are fits of the data based on the coupled oscillator model. For *D* = 40 nm, the upper HPP branch $E_{\text{HPP}}^{\text{U}}$ is outside of the measurement range. The horizontal gray dashed lines are the extracted resonance energies of the LSPR mode of the nanogrooves.

Figure 2 displays measured reflectance spectra versus the inverse grating period 1/*P* of the silver nanogroove gratings with depths *D* of 40 nm, 60 nm, 80 nm, and 100 nm, respectively, recorded for TM-polarization (electric field vector perpendicular to the nanogrooves). The spectra are normalized with respect to the reflectance of the bare silver flake. For *D* = 40 nm, the spectra show two bands of low reflectivity that we attribute to the lower and middle HPP branch. The corresponding upper HPP branch lies outside of the measurement range. In the case of the other nanogroove depths, the spectra feature three bands of low reflectivity that we associate with the three HPP branches. The dashed black curves are fits based on the coupled oscillator model introduced above. For each set, the spectral position of the lower HPP branch for the smallest grating period was used to determine the LSPR resonance energy *E*_LSPR_, while a linear fit of the middle HPP branch was used to determine *E*_SPP_(1/*P*). In the next step, the dispersion of the lower HPP branch was used to fit the coupling strength *g*. While the model nicely reproduces the dispersion of the lower and middle HPP branch, the agreement is less good for the upper HPP branch. This discrepancy can be attributed to some factors that are not included in the coupled oscillator model, e.g., the coupling of the LSPR mode to higher-order SPP modes and the effect of the first diffraction order of the nanogroove grating. As expected, the LSPR resonance energy *E*_LSPR_ shifts to lower energies as the nanogroove depth increases (40 nm nanogrooves: 2.91 eV; 60 nm nanogrooves: 2.65 eV; 80 nm nanogrooves: 2.4 eV; 100 nm nanogrooves: 2.17 eV). In contrast, the zero detuning energy splitting Δ*E*_UL_(0) between the upper and lower polariton branch is comparable for the four nanogroove geometries (40 nm nanogrooves: 676 meV; 60 nm nanogrooves: 769 meV; 80 nm nanogooves: 797 meV; 100 nm nanogrooves: 730 meV).

**Figure 3:**
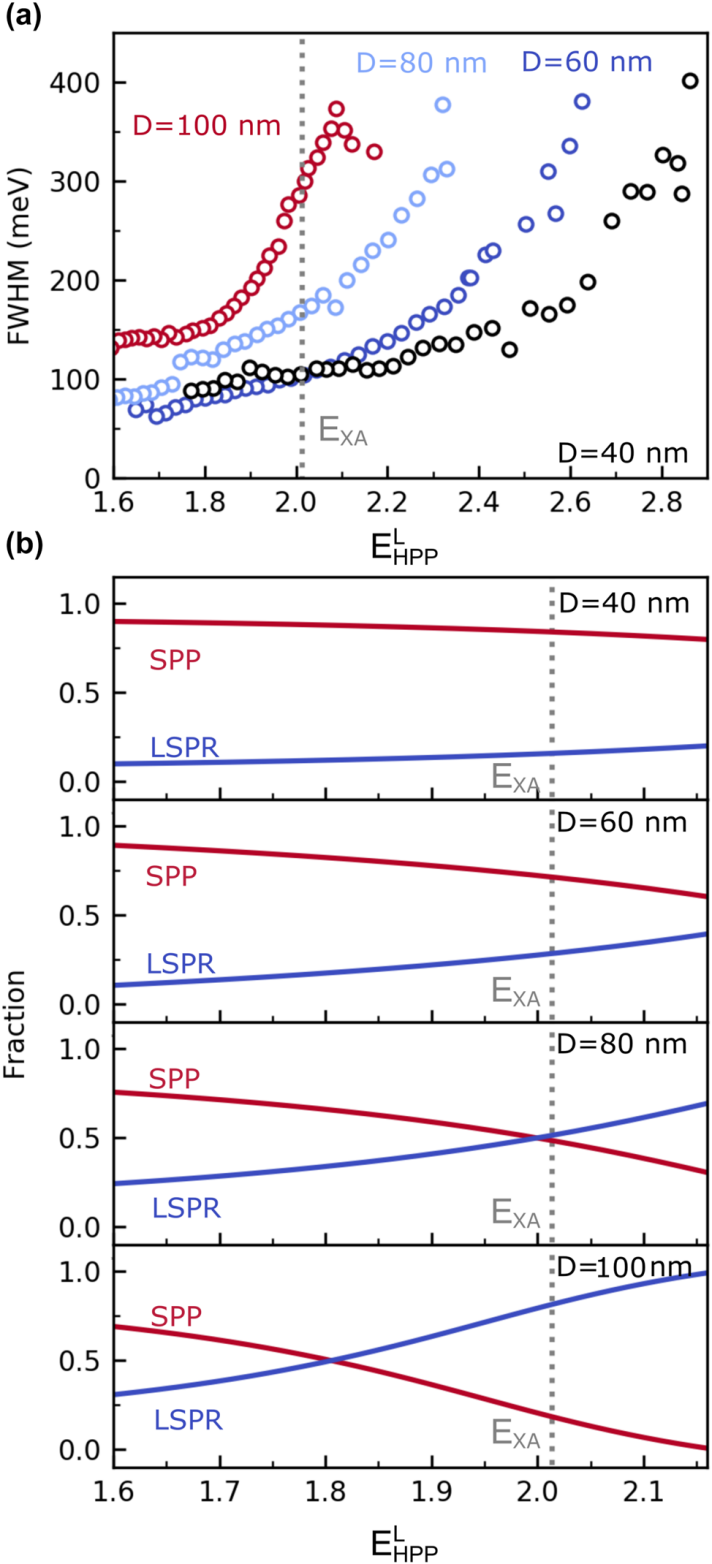
Properties of the lower HPP branch. (a) Fitted linewidths of the lower HPP branch of the four nanogroove grating structures. (b) SPP- (red) and LSPR-mode (blue) fractions of the lower HPP branch of the four nanogroove grating structures as derived from coupled oscillator models. The dashed gray line indicates the extracted A-exciton energy of the WS_2_ monolayer on a silver flake.

Figure 3(a) shows the fitted linewidths $\Gamma_{\text{HPP}}^{\text{L}}$ of the lower HPP branch of the four nanogroove grating structures. For all samples, we observe an increase of $\Gamma_{\text{HPP}}^{\text{L}}$ with $E_{\text{HPP}}^{\text{L}}$ . This trend is in each case a consequence of the varying LSPR (|*β*|^2^) and SPP (|*α* ^+^|^2^ + |*α* ^−^|^2^) fractions of the respective lower HPP branch (see Figure 3(b)). For $E_{\text{HPP}}^{\text{L}}\approx E_{\text{SPP}}$ , the lower HPP branch has a predominant SPP character and, hence, $\Gamma_{\text{HPP}}^{\text{L}}\approx \Gamma_{\text{SPP}}$ . As $E_{\text{HPP}}^{\text{L}}$ approches *E*_LSPR_, the LSPR fraction and, hence, also $\Gamma_{\text{HPP}}^{\text{L}}$ increases. For fixed energy, this results in an increasing linewidth with the nanogroove depth. For *D* = 60 nm and $E_{\text{HPP}}^{\text{L}}=E_{\text{xA}}=2.01\;eV$ , the lower HPP branch has a larger SPP fraction (|*α* ^+^|^2^ + |*α* ^−^|^2^ = 0.73, |*β*|^2^ = 0.27) resulting in an approximate linewidth of 104 meV. In the case of the 80 nm deep nanogrooves, the SPP and LSPR fractions are comparable at the A-exciton energy (|*α* ^+^|^2^ + |*α* ^−^|^2^ = 0.49, |*β*|^2^ = 0.51) leading to an increase in linewidth to 168 meV. Moreover, in the case of the 100 nm deep nanogrooves, the contribution of LSPR at the A-exciton energy is dominating (|*α* ^+^|^2^ + |*α* ^−^|^2^ = 0.22, |*β*|^2^ = 0.78) and the linewidth is approximately 300 meV. Unexpectedly, for *D* = 40 nm, the linewidth of the lower HPP branch is comparable to that of the 60 nm deep nanogrooves at the A-exciton energy despite of the larger SPP fraction (|*α* ^+^|^2^ + |*α* ^−^|^2^ = 0.84, |*β*|^2^ = 0.16). This can most likely be attributed to experimental limitations. Since both the numerical aperture of the microscope lens and the width of the adjustable slit of the spatial filter have nonzero values, the spectra are afflicted with spectral broadening induced by averaging over a finite range of incident angles as well as a small range of different grating periods.

**Figure 4:**
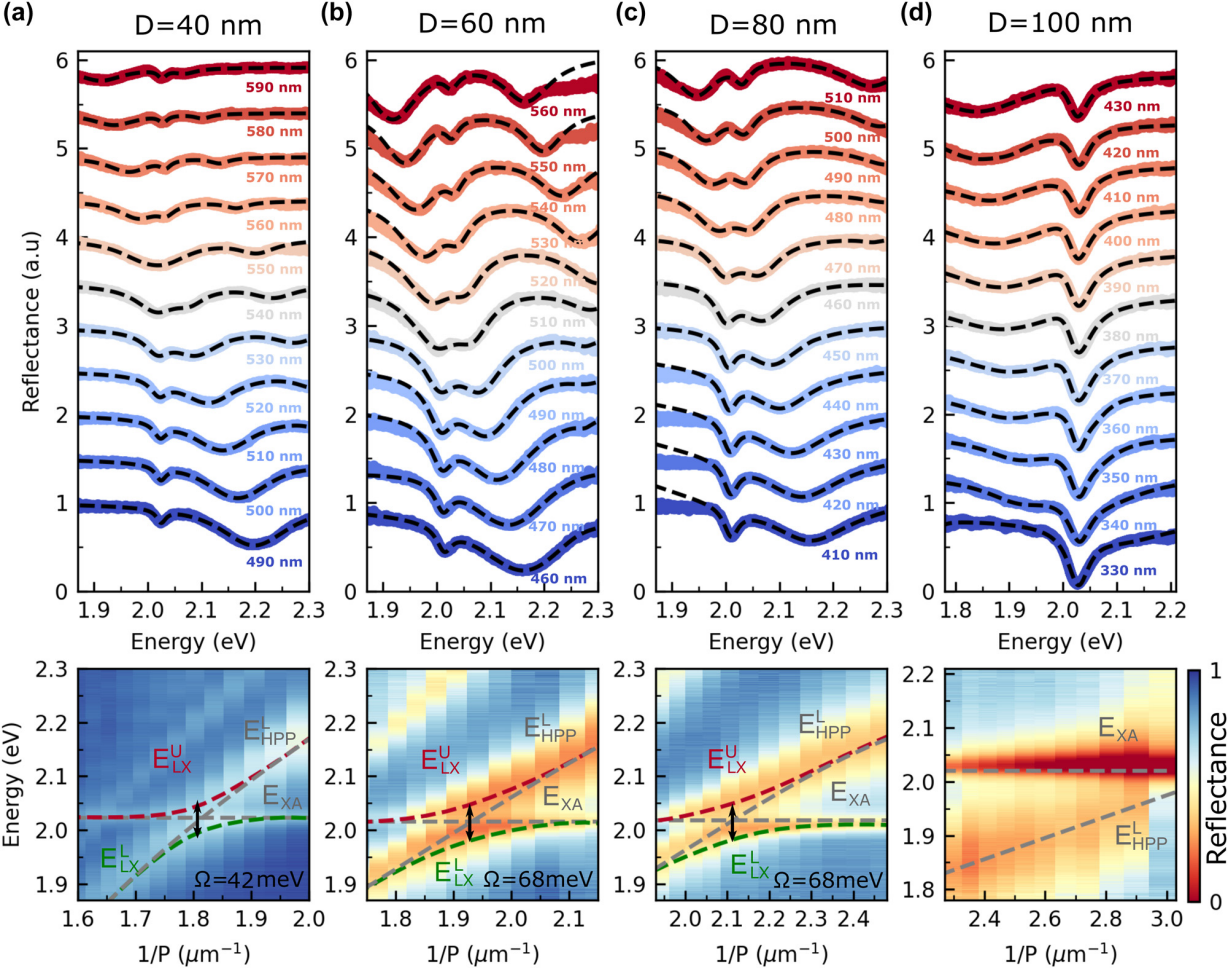
Reflectance spectra of the composite structures. (a)–(d) Normal incidence reflection spectra of WS_2_ monolayer coupled to nanogroove grating structures with 40 nm, 60 nm, 80 nm, and 100 nm depth, respectively, recorded for TM polarized light. The lower HPP branch is near-resonance with the A-exciton mode. The black dashed lines in the upper panels fit the spectra with the sum of the three Lorentzian line shapes. The red and green dashed lines in the lower panels depict the upper and lower polariton branches based on the coupled oscillator model. The tilted and horizontal gray dashed lines are extracted resonance energies of the lower HPP branch and the A-exciton mode.

Having characterized the individual components, we next discuss the properties of the composite structures. Figure 4(a) (top) depicts reflectance spectra of a WS_2_ monolayer deposited on the silver grating structure with 40 nm deep nanogrooves recorded with TM-polarized light. The spectra are normalized with respect to the reflectance of the bare silver flake. The chosen grating periods range between 490 nm and 590 nm, and the spectra are vertically offset for clarity. For *P* = 490 nm, the spectrum features two minima at 2.02 eV and 2.2 eV, which we identify as the A-exciton mode and the lower HPP branch, respectively. The latter shows a red shift of 56 meV compared to the bare nanograting case due to the increase of the local refractive index at the silver interface caused by the WS_2_ monolayer [45]. As the period increases, the lower HPP branch shifts to smaller energies. For *P* = 550 nm, the two modes merge in a broad dip without indication of an avoided crossing behavior. We note that a third reflection minimum observable for periods larger than 530 nm can be attributed to the middle HPP branch. A qualitatively different behavior can be observed in the case of the 60 nm and 80 nm deep nanogroove gratings (see Figure 4(b) and (c), top). For these samples, we observe a clear avoided crossing behavior of the two modes, indicating an appreciable interaction between the A-exciton and the lower HPP branch. Finally, for the 100 nm deep nanogrooves, the avoided crossing behavior is absent again (see Figure 4(d), top).

To model the interaction between the A-exciton mode and the lower HPP branch, we employ a coupled oscillator approach $\hat{H}{\left[\alpha_{HPP},\alpha_{XA}\right]}^{T}=E_{\text{LX}}{\left[\alpha_{HPP},\alpha_{XA}\right]}^{T}$ with the Hamiltonian

$$\hat{H}=\left(\begin{array}{cc} E_{\text{HPP}}^{\text{L}}-ı\frac{\Gamma_{\text{HPP}}^{\text{L}}}{2} & g_{\text{LX}}\\ g_{\text{LX}} & E_{\text{XA}}-ı\frac{\Gamma_{\text{XA}}}{2} \end{array}\right).$$

Here, *g*_LX_ is the coupling strength between the two modes. The Hopfield coefficients *α*_HPP_ and *α*_XA_ specify the fractions of the lower HPP branch and the A-exciton mode, respectively. The effect of the middle and upper HPP branch are ignored in this model because of their large detuning with respect to the A-exciton mode for the chosen grating periods. In contrast to the HPP formation discussed above, we cannot neglect the dampings when modeling the interaction of the A-exciton with the lower HPP branch, since $\Gamma_{\text{HPP}}^{\text{L}}$ and Γ_XA_ are comparable to *g*_LX_. The complex eigenenergies of the upper and lower branch of the coupled system are

$$E_{\text{LX}}^{\text{U,L}}=\frac{E_{\text{HPP}}^{\text{L}}+E_{\text{XA}}}{2}-ı\frac{\Gamma_{\text{HPP}}^{\text{L}}+\Gamma_{\text{XA}}}{4}\pm \frac{1}{2}\Delta E_{\text{UL}},$$

where $\Delta E_{\text{UL}}=2\sqrt{g_{\text{LX}}^{2}+\frac{1}{4}{\left[\delta_{\text{LX}}-\frac{ı}{2}\left(\Gamma_{\text{HPP}}^{\text{L}}-\Gamma_{\text{XA}}\right)\right]}^{2}}$ and $\delta_{\text{LX}}=E_{\text{HPP}}^{\text{L}}-E_{\text{XA}}$ denotes the detuning between the lower HPP branch and the A-exciton mode. The energy difference between the upper and the lower branch for *δ*_LX_ = 0, i.e., the Rabi splitting Ω, is given by $\Omega =2\sqrt{g_{\text{LX}}^{2}-\frac{1}{16}{\left[\Gamma_{\text{HPP}}^{\text{L}}-\Gamma_{\text{XA}}\right]}^{2}}$ . For *g*_LX_ > (Γ_LSL_ − Γ_XA_)/4, Ω is real and a mode splitting occurs. However, this splitting can be indistinguishable from an induced transmission due to Fano interference resulting from the spectral interference of the narrow exciton resonance with a broad plasmon resonance [46]. Hence, the accepted criterion to identify the strong coupling regime is that at least one Rabi oscillation must occur, which requires *g*_LX_ > *g*_c_ = (Γ_LSL_ + Γ_XA_)/4 [47], [48], [49], [50].

To extract the coupling strength *g*_LX_ from the experimental data, we use the following procedure. We determine the energies of the coupled modes of the WS_2_ nanograting composite structure by fitting each reflection spectrum shown in Figure 4 (top) with a sum of three Lorentzian lines (including one for the middle branch). We then calculate for each period the energy difference of the two extracted mode energies. The smallest energy difference corresponds to *δ*_LX_ = 0 and is taken as the Rabi energy Ω. Together with the linewidth Γ_XA_ of the bare WS_2_ monolayer (see Figure 1(e)) and the linewidth $\Gamma_{\text{HPP}}^{\text{L}}$ of the nanograting structure at the exciton energy (see Figure 2(a)), the coupling strength follows from

(1) $$g_{\text{LX}}=\sqrt{\frac{\Omega^{2}}{4}+\frac{1}{16}{\left[\Gamma_{\text{HPP}}^{\text{L}}-\Gamma_{\text{XA}}\right]}^{2}}.$$

The dashed red and green lines superimposed on the color-coded reflectance spectra shown in Figure 4 (bottom) are the dispersion of the upper and lower polariton branch as predicted by the coupled oscillator model.

For the composite structure with the 40 nm deep nanogrooves (see Figure 4(a)), we extract from the Lorentzian fits to the reflection spectra a Rabi splitting of 42 meV. Together with $\Gamma_{\text{HPP}}^{\text{L}}=104\;meV$ and Γ_XA_ = 35 meV, we obtain a coupling strength *g*_LX_ of 30 meV. In comparison, the critical coupling strength *g*_c_ for the given linewidths is 35 meV. This indicates, that for this composite structure, the lower HPP branch is only weakly coupled to the A-exciton mode of the monolayer.

When the WS_2_ monolayer is coupled to the 60 nm deep nanogrooves (see Figure 4(b)), the Rabi splitting is 68 meV corresponding to a coupling strength *g*_LX_ of 38 meV. The increase in the coupling strength can be explained by a tighter field confinement due to the higher LSPR fraction of the lower HPP branch. Since the linewidths $\Gamma_{\text{HPP}}^{\text{L}}$ and Γ_XA_ and, thus, also the critical coupling strength *g*_c_ are the same as in the previous case, we find that the strong coupling condition *g*_LX_ > *g*_c_ is fulfilled.

Next, we discuss the interaction of a WS_2_ monolayer with the silver grating structure with 80 nm deep nanogrooves (see Figure 4(c)). On the one hand, we anticipate that the growing LSPR fraction of the lower HPP branch leads to a further increase of the coupling strength *g*_LX_. On the other hand, the increasing linewidth of the lower HPP will cause a larger critical coupling strength *g*_c_. The Rabi splitting Ω extracted from the experimental data is 68 meV. Together with $\Gamma_{\text{HPP}}^{\text{L}}=168\;meV$ and Γ_XA_ = 35 meV, this results in a coupling strength *g*_LX_ of 48 meV. As expected, this value is larger than in the previous two cases. At the same time, however, the critical coupling strength *g*_c_ rises to 51 meV due to an increase of the linewidth of the lower HPP branch (see discussion of Figure 3). As a result, the composite structure with the 80 nm deep nanogrooves does not meet the strong coupling criterion, i.e., the increase of the damping dominates over the increase of the coupling strength. This example demonstrates that it is not sufficient to observe an avoided crossing to claim strong coupling.

One might now speculate that the situation could be reversed when the detuning between the LSPR mode and the A-exciton mode is further reduced. However, the reflectance spectra of the composite structure with 100 nm deep nanogrooves show that this does not happen (see Figure 4(d) (top)). The spectra feature two minima, a pronounced exciton resonance dip at 2.02 eV and a shallow dip caused by the lower HPP branch that shifts to higher energy with decreasing period. As the lower HPP branch approaches the A-exciton, we do not observe an avoided crossing of the modes (also see Figure 4(d) (bottom)) and, hence, no indication for strong coupling.

## 3 Conclusions

In summary, we have studied light–matter interactions at room temperature in composite structures formed by coupling of WS_2_ monolayers to different silver nanogroove gratings. The latter support hybrid plasmon polaritons formed by strong coupling of localized surface plasmon resonances (LSPR) in the nanogrooves with propagating surface plasmon polaritons (SPP) on the silver film. By increasing the nanogroove depth from 40 nm to 100 nm, the character of the lower branch of the hybrid plasmon polariton at the energy of the WS_2_ A-exciton mode changes from SPP-like to LSPR-like. This transition is accompanied by an increasing field confinement and a significant growth of the damping. For appropriate nanogroove depths, these two counteracting effects can be balanced. Using reflection spectroscopy, we demonstrate strong coupling between the A-exciton mode of the WS_2_ monolayer and the lower HPP branch of the nanograting structure with 60 nm deep nanogrooves. In contrast, the strong coupling condition is not met for shallower or deeper nanogratings since either the field confinement is not sufficient or the damping of the HPP mode is too large.

We envision that those hybrid plasmon polaritons coupled to TMDC monolayers also hold great prospects for future investigations. For instance, dark excitons in TMDC monolayers with zero in-plane transition dipole moment are challenging to detect with conventional far-field optical techniques but can be probed by near-field coupling to SPP modes [51]. The opportunity to tailor the field strength and distribution of the hybrid plasmon polaritons could be used in future experiments to tailor their interaction with dark excitons leading to efficient extraction of the dark exciton emission. Moreover, the potential to tailor the character of the HPP could also be interesting for the efficient transfer of hot electrons into the TMDC monolayer [39].

## 4 Methods

### 4.1 Synthesis of crystalline silver flakes

Monocrystalline silver crystals were synthesized based on an ammonium hydroxide-controlled polyol reduction process [41]. Firstly, we prepared 15 ml of a 0.17 M silver nitrate (Sigma Aldrich) ethylene glycol (EG) (Sigma Aldrich) solution. Then, ammonium hydroxide solution was added (28–30 %, Sigma Aldrich, 1.85 ml) to stabilize the reaction. In addition, polyvinylpyrrolidone (*Mw* = 55 k, 0.5 g), which acts as a capping agent, was added to slow down the dispersion as well as the rate of growth. Furthermore, chloroplatinic acid hydrate (H2PtCl6 ⋅ xH2O, > 99.995, Sigma Aldrich, 0.54 mL of 0.02M in water) was added to form platin nanoparticles, which serve as nucleation centers for the silver atoms. Finally, hydrogen peroxide (30 %, Chemsolute 1.3 mL) was added to start the reduction of the silver salt. The substrates are cleaned and added into the growth container before the mixture is added. Then the growth solution is left for several days. So the substrates are in the solution the whole time. After the growth, the substrates were cleaned with distilled water to remove excess chemicals. The crystalline silver flakes prepared in this way exhibit an excellent surface quality with a root mean square roughness of less than 0.5 nm as determined by atomic force microscopy.

### 4.2 Fabrication of nanogroove grating structures

Fan-shaped nanogroove gratings were fabricated by focus ion beam (FIB) lithography. The system is based on a Zeiss 1540XB Crossbeam microscope with a gallium (Ga^2+^) ion source and a Raith Elphy nanopatterning system. An ion beam with an acceleration voltage of 30 keV and a current of approximately 50 pA was used in combination with a 30 µm aperture. The relatively high current helps to reduce the patterning time to below 15 min. The nanogroove depth was determined by tilted view electron microscopy of cross sections of the gratings prepared by FIB milling. Additionally, we employed atomic force microscopy to check the uniformity of the nanogroove gratings. To prevent the grating from deteriorating due to silver oxidation, the nanogroove gratings were stored in a vacuum environment, and the measurement was completed within several days after grating fabrication.

### 4.3 Preparation of WS_2_ monolayers

Atomically thin WS_2_ monolayers were fabricated by a mechanical exfoliation method [42] from a WS_2_ crystal (2D Semiconductors). Monolayers were identified by micro differential reflectance spectroscopy [52], [53]. The WS_2_ monolayer was firstly deposited on a PDMS stamp and then transferred onto the nanograting structure. The WS_2_ monolayers prepared in this way have typical dimensions of several ten micrometers.

## Supplementary Material

Supplementary Material Details
