# Supplementary material for: Strong coupling between WS2 monolayer excitons and a hybrid plasmon polariton at room temperature
Source: Nanophotonics. 2024 Apr 15;13(15):2847–56. doi: 10.1515/nanoph-2024-0021 (PMC11502097; doi:10.1515/nanoph-2024-0021)
Supplement: Supplementary file 1 — Supplementary Material Details [file j_nanoph-2024-0021_suppl_001.pdf]

Supplementary Material: Strong coupling between WS_2 monolayer excitons and a hybrid plasmon polariton at room temperature.

Yuhao Zhang, Hans-Joachim Schill, Stephan Irsen, Stefan Linden

1 Dielectric function of WS_2 monolayers

To determine the dielectric function $\epsilon(\mathrm{E})$ of a WS_2 monolayer, the transmission spectrum of a WS_2 monolayer on a PDMS stamp (see Fig. S1) was fitted using the transfer matrix method and a multi-Lorentzian model:

$$\epsilon(\mathrm{E}) = 1 + \sum_{\mathrm{j}=1}^{5} \frac{\mathrm{f_j}}{\mathrm{E_j^2} - \mathrm{E^2} - \mathrm{iE}\Gamma_\mathrm{j}} \tag{1}$$

Here, E is the photon energy in eV. $\mathrm{f_j}$, $\mathrm{E_j}$, and Γ_j are the oscillator strength, the resonance energy, and the damping constant of the j-th resonance. The thickness of the monolayer is set at 0.6 nm. The parameters resulting from the fitting procedure are summarized in Table S1. Figure S1 b depicts the real and imaginary part of the refractive index $n(E) = \sqrt{\epsilon(\mathrm{E})}$ of a WS_2 monolayer.

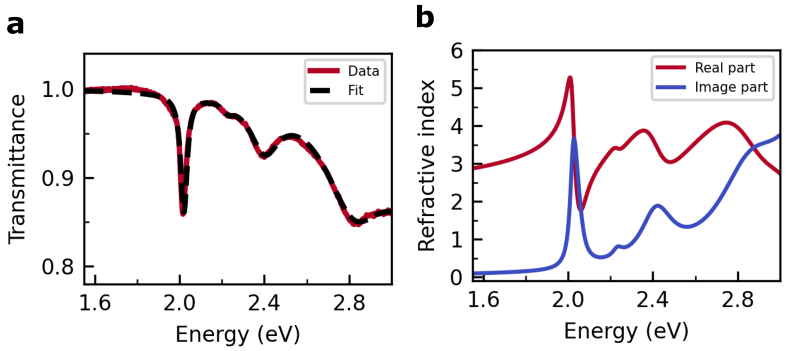

Figure S1: (a) Measured and calculated transmission spectra of a WS_2 monolayer on a PDMS stamp. (b) Real and imaginary part of the refractive index of WS_2 monolayer.

Table S1: WS_2 monolayers dispersion parameters.

Oscillator No. j	Oscillator strength $\mathrm{f_j}$	Resonance energy $\mathrm{E_j}$ (eV)	Damping constant Γ_j (eV)
1	1.778	2.020	0.029
2	0.255	2.221	0.028
3	2.445	2.402	0.122
4	9.879	2.831	0.237
5	40.539	3.093	0.424

2 Calculated reflectance spectra of silver nanogroove gratings

Normal incidence reflectance spectra of silver nanogroove gratings were numerically calculated using the finite element method (Comsol Multiphysics). In these calculations, we considered a 2D computational domain with periodic boundary conditions along the x-direction. The spectra were calculated for TM polarized light, i.e., the electric field vector of the incident plane wave was oriented in x-direction. For the dielectric function of silver, we used the experimental data of Johnson and Christy [1]. We assumed that the nanogooves have a Gaussian shape with a width of 100 nm.

Figure S2 shows calculated reflectance spectra of silver nanogroove gratings versus the inverse grating period $1/P$ for nanogroove depths ranging from 40 nm to 100 nm. In contrast to the experimental data presented in the article, the calculated spectra feature only two bands of low reflectance. This is expected, since the middle HPP branch can not be excited with a plane wave at normal incidence for symmetry reasons [2]. The dashed black lines are fits of the oscillator model to the reflectrance spectra. The calculated dispersion of the lower HPP branch shows similar trends as in the experiments. With increasing nanogroove depths, slope of the lower HPP branch decreases since due to the red shift of the LSPR mode. At the same time, the linewidth of the lower HPP branch $\Gamma^{\mathrm{L}}_{\mathrm{HPP}}$ increases (see Fig. S2 e).

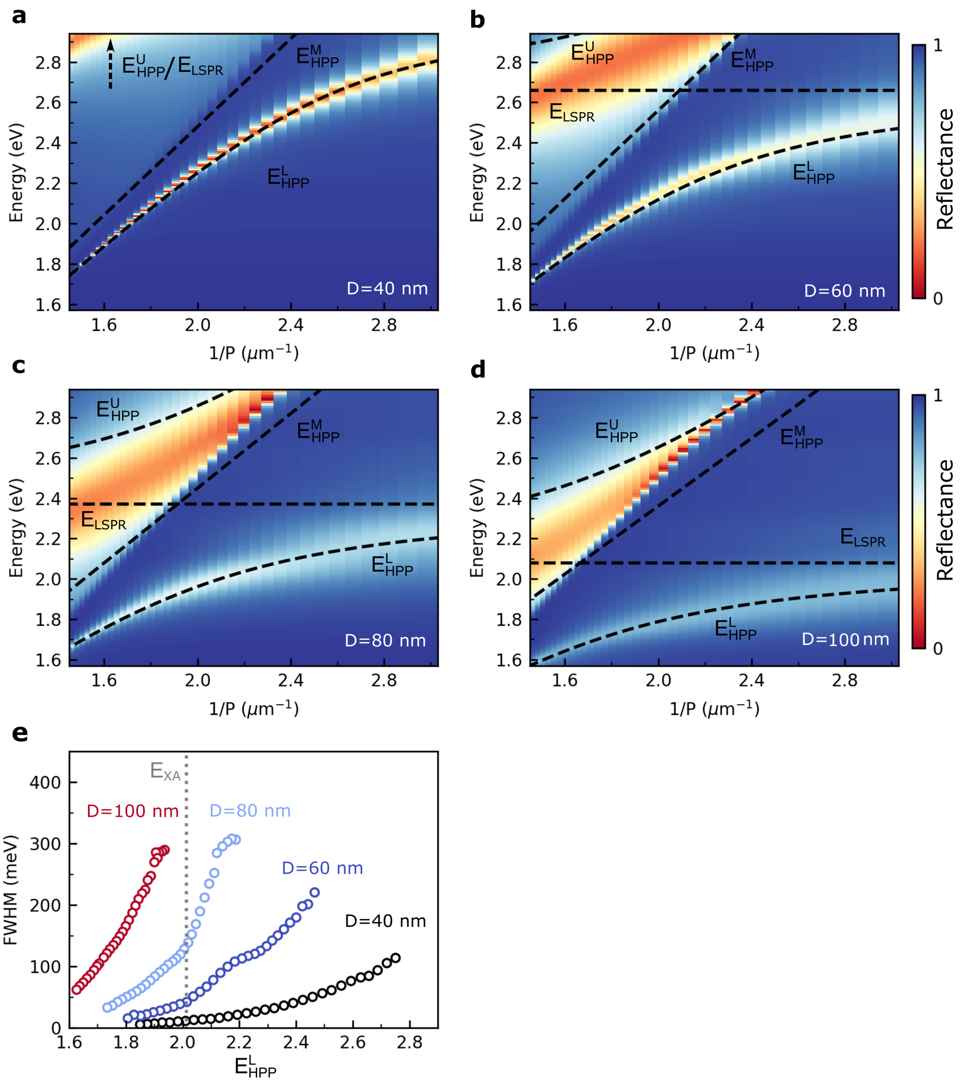

Figure S2: (a-d) Color-coded normal incidence spectra versus the inverse grating period $1/P$ of nanogroove grating structures with nanogroove depths D of 40 nm, 60 nm, 80 nm and 100 nm, respectively. The spectra were calculated for TM polarized incident light. The dashed black curves are fits based on the coupled oscillator model. For $D = 40\,\mathrm{nm}$, the upper HPP branch $\mathrm{E}^{\mathrm{U}}_{\mathrm{HPP}}$ is outside of the considered spectral range. (e) Linewidth of the lower HPP branch of the four nanogroove grating structures.

3 Calculated reflectance spectra of composite structures consisting of a WS_2 monolayer and a silver nanogroove grating

We used the finite element method to calculate reflectance spectra of composite structures consisting of a WS_2 monolayer and a silver nanogroove grating. The WS_2 monolayer was implemented as a transition boundary condition on the surface of the silver film with a thickness of 0.6 nm and the refractive index shown in section 1. Figure S3 depicts calculated reflectance spectra versus the inverse grating period $1/P$ of composite structures with nanogroove depths ranging from 40 nm to 100 nm.

In the following discussion, we concentrate on the spectral range around the A-exciton energy (2.02 eV). The composite structures with 40 nm and 60 nm deep nanogrooves show an avoided crossing behavior between the lower HPP branch and the A-exciton mode. The derived coupling strength g_c is 13 meV for $D = 40\,\mathrm{nm}$ and 23 meV for $D = 60\,\mathrm{nm}$. These values are smaller than the experimentally ones indicating that the coupling strength is underestimated in the calculations. Since the calculated linewidth of the lower HPP is in each case smaller than the corresponding experimental value, the two structures nevertheless fulfill the strong coupling condition. In contrast, no avoided crossing is observed for the composite structures with 80nm and 100 nm deep nanogrooves.

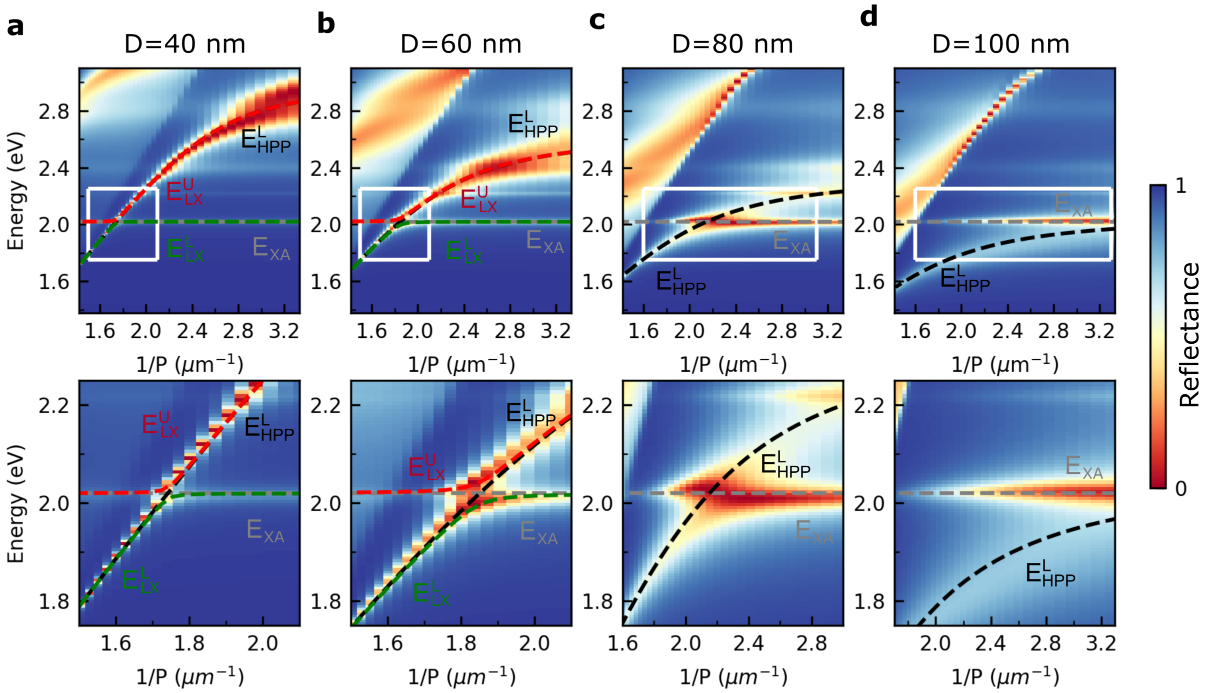

Figure S3: (a-d) Color-coded normal incidence spectra versus the inverse grating period $1/P$ of composite structures with nanogroove depths D of 40 nm, 60 nm, 80 nm and 100 nm, respectively. The spectra were calculated for TM polarized incident light. The red and green dashed lines indicate the upper and lower polariton branches based on the coupled oscillator model used to model the coupling of the lower HPP branch and the A-exciton resonance. Lower panel: Detailed spectra corresponding to the region of the white rectangles depicted in the respective upper panel.

References

[1] P. B. Johnson and R. W. Christy, "Optical constants of the noble metals," *Phys. Rev. B*, vol. 6, p. 4370, 1972.

[2] C.-Y. Wang, Y. Sang, X. Yang, S. S. Raja, C.-W. Cheng, H. Li, Y. Ding, S. Sun, H. Ahn, C.-K. Shih, S. Gwo, and J. Shi, "Engineering giant rabi splitting via strong coupling between localized and propagating plasmon modes on metal surface lattices: Observation of $\sqrt{N}$ scaling rule," *Nano Letters*, vol. 21, pp. 605–611, 12 2020.
